# The role for ambulatory electrocardiogram monitoring in the diagnosis and prognostication of Brugada syndrome: a sub-study of the Rare Arrhythmia Syndrome Evaluation (RASE) Brugada study

**DOI:** 10.1093/europace/euae091

**Published:** 2024-04-08

**Authors:** Chiara Scrocco, Yael Ben-Haim, Bode Ensam, Robert Aldous, Maite Tome-Esteban, Mark Specterman, Michael Papadakis, Sanjay Sharma, Elijah R Behr

**Affiliations:** Cardiovascular Clinical Academic Group St. George’s, University of London and St. George’s University Hospitals NHS Foundation Trust, Cranmer Terrace, London SW17 0RE, UK; Cardiovascular Clinical Academic Group St. George’s, University of London and St. George’s University Hospitals NHS Foundation Trust, Cranmer Terrace, London SW17 0RE, UK; Cardiovascular Clinical Academic Group St. George’s, University of London and St. George’s University Hospitals NHS Foundation Trust, Cranmer Terrace, London SW17 0RE, UK; Cardiovascular Clinical Academic Group St. George’s, University of London and St. George’s University Hospitals NHS Foundation Trust, Cranmer Terrace, London SW17 0RE, UK; Cardiovascular Clinical Academic Group St. George’s, University of London and St. George’s University Hospitals NHS Foundation Trust, Cranmer Terrace, London SW17 0RE, UK; Cardiovascular Clinical Academic Group St. George’s, University of London and St. George’s University Hospitals NHS Foundation Trust, Cranmer Terrace, London SW17 0RE, UK; Cardiovascular Clinical Academic Group St. George’s, University of London and St. George’s University Hospitals NHS Foundation Trust, Cranmer Terrace, London SW17 0RE, UK; Cardiovascular Clinical Academic Group St. George’s, University of London and St. George’s University Hospitals NHS Foundation Trust, Cranmer Terrace, London SW17 0RE, UK; Cardiovascular Clinical Academic Group St. George’s, University of London and St. George’s University Hospitals NHS Foundation Trust, Cranmer Terrace, London SW17 0RE, UK

**Keywords:** Brugada syndrome, ECG, Holter monitoring, Sudden death

## Abstract

**Aims:**

Brugada syndrome (BrS) diagnosis and risk stratification rely on the presence of a spontaneous type 1 (spT1) electrocardiogram (ECG) pattern; however, its spontaneous fluctuations may lead to misdiagnosis and risk underestimation. This study aims to assess the role for repeat high precordial lead (HPL) resting and ambulatory ECG monitoring in identifying a spT1, and evaluate its prognostic role.

**Methods and results:**

HPL resting and ambulatory monitoring ECGs of BrS subjects were reviewed retrospectively, and the presence of a spT1 associated with ventricular dysrhythmias and sudden cardiac death (SCD). Three-hundred and fifty-eight subjects (77 with spT1 pattern at presentation, Group 1, and 281 without, Group 2) were included. In total, 1651 resting HPL resting and 621 ambulatory monitoring ECGs were available for review, or adequately described. Over a median follow-up of 72 months (interquartile range - IQR - 75), 42/77 (55%) subjects in Group 1 showed a spT1 in at least one ECG. In Group 2, 36/281 subjects (13%) had a newly detected spT1 (1.9 per 100 person-year) and 23 on an HPL ambulatory recording (8%). Seven previously asymptomatic subjects, five of whom had a spT1 (four at presentation and one at follow-up), experienced arrhythmic events; survival analysis indicated that a spT1, either at presentation or during lifetime, was associated with events. Univariate models showed that a spT1 was consistently associated with increased risk [spT1 at presentation: hazard ratio (HR) 6.3, 95% confidence interval (CI) 1.4–28, *P* = 0.016; spT1 at follow-up: HR 3.1, 95% CI 1.3–7.2, *P* = 0.008].

**Conclusion:**

Repeated ECG evaluation and HPL ambulatory monitoring are vital in identifying transient spT1 Brugada pattern and its associated risk.

## Graphical Abstract

**Graphical Abstract:**
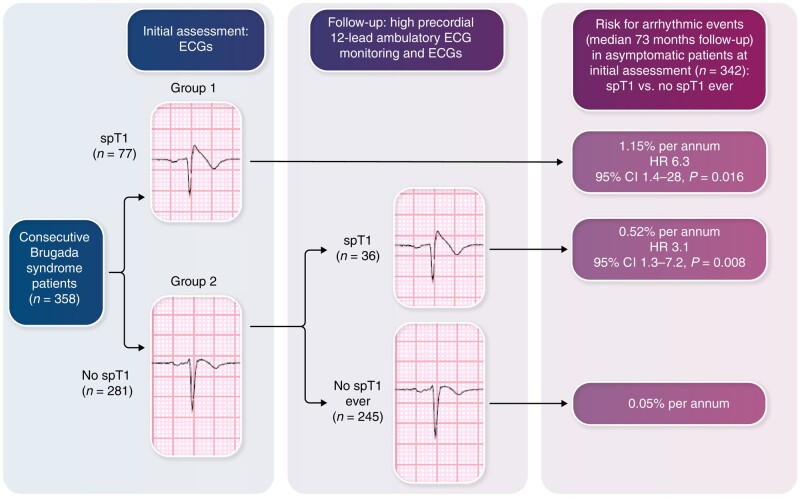
The implications of high precordial 12-lead resting and ambulatory ECG monitoring for Brugada syndrome: the flow diagram indicates the patient cohort and selection according to the presence of a spontaneous type 1 (spT1) Brugada pattern on the ECG at initial assessment (Group 1) and then subsequently during follow-up with high precordial 12-lead resting and ambulatory ECG monitoring (Group 2). Arrhythmic event rates and hazard ratios (HRs) are given for asymptomatic patients from each group with a spT1 vs. never showing a spT1 at all.

**What’s new?**

- Follow-up evaluation with 24-h 12-lead ambulatory ECG monitoring using the high right precordial leads is able to identify the dynamic type 1 Brugada ECG pattern in patients without a spontaneous type 1 Brugada ECG pattern on their ECGs at first presentation.
- The discovery of a spontaneous type 1 Brugada ECG pattern on 24-h 12-lead ambulatory ECG monitoring during follow-up is associated with an increased risk of events.
- This has important implications for how patients with concealed Brugada syndrome should be optimally managed and followed up for risk stratification.

## Introduction

The Brugada syndrome (BrS) is an inherited arrhythmogenic condition that can predispose to fatal ventricular tachyarrhythmias and has also been associated with ventricular standstill and heart block.^1–3^ Current guidelines recommend that BrS can be diagnosed in the presence of an unprovoked spontaneous type 1 (spT1) pattern in lead V1 or V2 on the surface electrocardiogram (ECG) positioned at the sternal margins at the fourth intercostal space (ICS), or at the third or second intercostal spaces (the ‘high precordial leads’—HPLs). The type 1 pattern is characterized by coved ST-segment elevation ≥2 mV and a negative T-wave and may be concealed and only induced (diT1) after provocative drug testing with intravenous sodium channel blockers (SCBs). In this case, a diagnosis of ‘concealed’ BrS may be corroborated by previous aborted cardiac arrest (aCA), arrhythmic syncope or nocturnal agonal respiration, and/or a family history of BrS or of juvenile sudden death (SD) with a negative autopsy and circumstance suspicious for BrS.^4^ Several clinical and ECG parameters have been proposed to identify subjects at risk of experiencing life-threatening arrhythmias during follow-up^5–7^; a spT1 pattern at presentation has consistently been associated with increased risk, whereas a diT1 confers a better prognosis; however, the Brugada ECG is highly variable over time,^8,9^ and therefore, relying only on the resting 12-lead ECG at presentation may underestimate the actual risk. The use of 12-lead 24-h ambulatory ECG monitoring with V1–V2 simultaneously recorded in the standard and HPL configuration (HPL Holter) has been shown to increase the detection of a new transient spT1.^10–12^ But the correlation between spT1 appearance at follow-up and arrhythmic events (AEs) has not been investigated so far. The aim of this retrospective analysis was two-fold: (i) to systematically assess the yield of repeat resting and HPL 12-lead ECGs and the additional role of HPL Holter recordings in identifying a new spT1 and the clinical and ECG predictors of its appearance and (ii) to evaluate the prognostic role of spT1 pattern recorded in such a manner in a large cohort of BrS patients.

## Methods

### Study population

The Rare Arrhythmia Syndrome Evaluation (RASE) consortium is a collection of collaborators from the UK established in 2016 aiming to conduct observational and interventional studies in the arrhythmia syndromes coupled with longitudinal follow-up. The RASE-Brugada registry consecutively recruited subjects with a diagnosis of BrS as per consensus guidelines at the time of enrolment. The study was approved by the regional ethics committee (South West London Rec 3) and local Trusts R&D Institutes, and all patients gave their informed consent to the inclusion in the research. Exclusion criteria included evidence of significant coronary disease (>70% stenosis in at least one coronary artery, or >50% stenosis of the left main, and/or ischaemia on a functional test); evidence of significant cardiomyopathic disease (outside normal range ventricular function and structure on echocardiography and/or cardiac magnetic resonance imaging); and metabolic abnormality at time of a type 1 ECG pattern (e.g. hyperkalaemia or hypercalcaemia).

### Clinical evaluation

For all subjects, demographic, genetic, historical clinical, and follow-up data were collected. Patients were classified as symptomatic if they had a history of aCA, documented ventricular tachycardia (VT), or clearly arrhythmic syncope. A subject was classified as having a spT1 if this was present on the ECG at presentation. A proband was defined as the first member of a family being evaluated or diagnosed. A positive genotype was defined as the presence of a pathogenic or likely pathogenic variant in the *SCN5A* gene according to current variant interpretation criteria.^13^ All patients were advised to avoid the medications known to potentially elicit a spT1 and to treat high temperature promptly as per current guidelines and received regular follow-up, usually on an annual basis. Subjects enrolled in the RASE-Brugada registry were included in this study who had complete clinical data, at least 3-month follow-up data, and a minimum of two 12-lead ECGs (either standard or HPL) or one 12-lead ECG and one HPL Holter recording. Follow-up duration comprised the time from first clinical evaluation to 31 December 2021, the date of an AE [defined as sudden cardac death (SCD), appropriate implantable cardioverter defibrillator (ICD) intervention or documented polymorphic VT (pVT), or ventricular standstill], or the start of pharmacological treatment with quinidine, whichever came first.

### Resting and ambulatory electrocardiogram recording and analysis

All subjects underwent resting and HPL 12-lead ECG recording at presentation and at regular follow-up visit. Electrocardiograms were recorded at a paper speed of 25 mm/s and amplitude of 10 mm/mV. High precordial lead Holter recordings were recorded using the CardioMem® CM 3000-12 BT recorder (sample rate 1024s/s, Getemed, GE Healthcare) and H12+ Digital recorder (sample rate 1000s/s, Mortara Instruments, Baxter). Electrodes exploring leads V1–V2 were positioned at the fourth and third ICS and, from June 2018, also in the second ICS. All resting and Holter ECGs were reviewed manually to adjudicate the presence of a spT1. When a tracing was not available for review but described in the medical records by a senior cardiologist with expertise in inherited cardiac conditions (E.R.B.), this was included in the analysis.

### Statistical analysis

Data showing a normal distribution, checked via the Shapiro–Wilk test, are presented as means ± standard deviation (SD), otherwise as median and interquartile range (IQR). Continuous variables were analysed using Student’s *t*-test or the Mann–Whitney *U* test, whereas categorical variables with the *χ*^2^ test or Fisher’s exact test. Survival curves were plotted by means of the Kaplan–Meier method and compared using the log-rank test. Cox proportional hazards regression models were used to estimate the effect of the risk factor (the presence of a spT1) on the outcome. A *P* < 0.05 was considered statistically significant. Statistical analyses were performed using IBM®SPSS® Statistics v.29 software.

## Results

**Figure 1:**
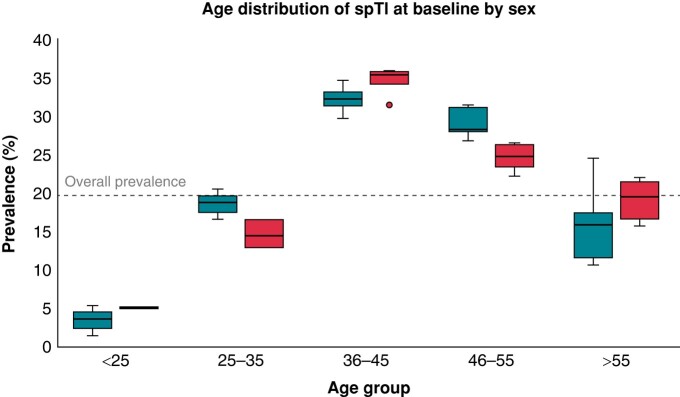
Age distribution at spontaneous type 1 at baseline evaluation by sex. Turquoise plot box represents males, and red plot box represents females. Overall prevalence of spontaneous Type 1 pattern (spT1) at presentation was 21% (grey dotted line). Median age at presentation was 44 [IQR 17] years in males and 45 [IQR15] years in females (*P* = not significant).

A total of 358 subjects consecutively evaluated at St George’s University Hospitals NHS Foundation Trust, London, UK, from June 2002 to December 2021 were included in the study. Seventy-seven showed a spT1 at presentation (Group 1), whereas 281 did not (Group 2). In the former group, 11 patients had a spT1 recorded during fever and 1 during an Addisonian crisis without reported electrolyte derangement. *Figure 1* depicts the age and sex distribution of Group 1. In Group 2, all patients underwent pharmacological testing with SCB, which unmasked a diT1 pattern, aside from six; of these, five showed a spT1 after presentation but before undergoing the SCB test, and in one subject, a diT1 pattern was elicited by antiarrhythmic therapy with flecainide for atrial fibrillation. *Table 1* details the demographic, clinical, genetic, and follow-up data of the two groups. The median age at presentation was 44 ± 15 years, and the majority of subjects were of white ethnicity. Subjects with a spT1 pattern at presentation were more frequently males and probands and more likely to have experienced syncope or pre-syncope compared to those with a concealed T1; conversely, they were less likely to have a personal history of aCA/documented pVT or a family history of SD. The overall prevalence of pathogenic/likely pathogenic variants in the *SCN5A* gene was 25%, without differences in the two groups.

**Table 1:** Summary of demographic and clinical characteristics of Brugada syndrome patients with a spontaneous (spT1) vs. concealed type 1 pattern at presentation

	Spontaneous type 1 at presentation, *N* = 77	Concealed type 1, *N* = 281	*P* value
Male gender (%)	57 (74)	127 (45)	**<0.001**
White ethnicity	51 (66)	215 (76)	NS
Age at presentation	45 [17]	44 [26]	NS
Proband status	72 (94)	164 (58)	**<0.001**
P/LP variant in *SCN5A* gene	18/55 (33)	32/147 (22)	NS
Prior symptoms			
** **aCA/VT	0^a^	14^b^	NS
** **Syncope/pre-syncope	32 (42)	69 (25)	**0.003**
** **Palpitations	15 (19)	59	NS
Family history of SD	15/74 (20)	153/273 (56)	**<0.001**

Values are expressed as median with IQR or absolute number with percentage. Bold values are only for significant *P* values.

^a^Two subjects had ventricular arrhythmias recorded.

^b^One subject had nocturnal agonic respiration.

aCA, aborted cardiac arrest; P/LP, pathogenic/likely pathogenic; SD, sudden death; VT, ventricular tachycardia.

### Electrocardiogram and high precordial lead Holter recording analysis

**Figure 2:**
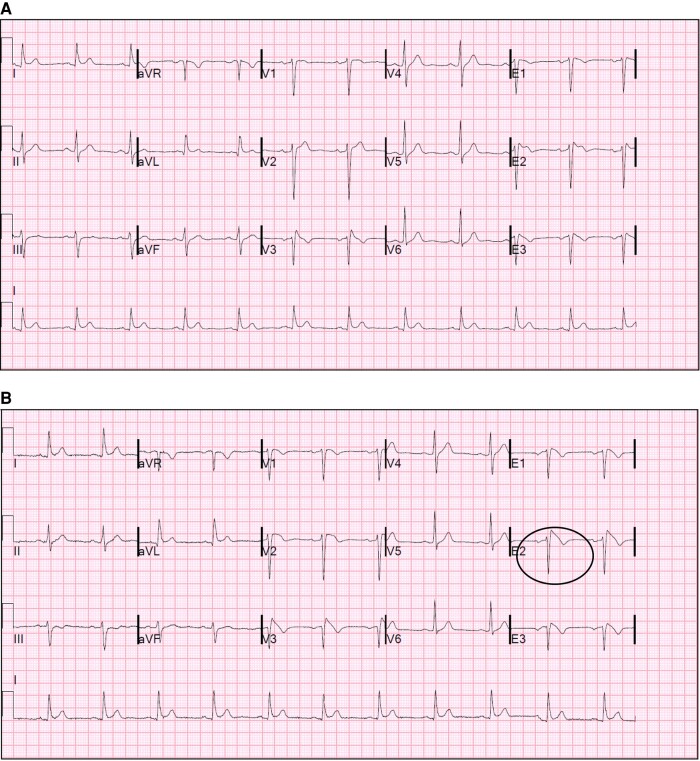
A 58-year-old Caucasian male with baseline high precordial electrocardiogram (ECG) (*A*) and dynamic spontaneous T1 pattern recorded at follow-up ECG (*B*). In the left panel, leads V3–V6 are recorded in the third and second intercostal spaces; in the right panel, V3 is lead V1 recorded in the third intercostal space, E1 is V2 recorded in the third intercostal space, E2 is V1 recorded in the second intercostal space, and E3 is V2 recorded in the second intercostal space.

**Figure 3:**
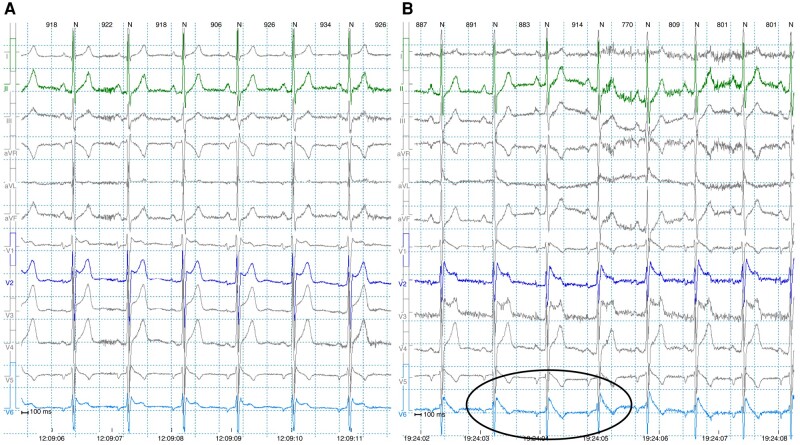
A 52-year-old Caucasian male with transient spontaneous type 1 Brugada electrocardiogram (ECG) pattern appearing during 12-lead high precordial 24-h ambulatory ECG monitoring (*A*) compared to another time extract from the same recording (*B*). In this recording, leads V1 and V2 are recorded in the fourth intercostal space; V3 and V4 are V1 and V2 recorded in the third intercostal space; and V5 and V6 are V1 and V2 recorded in the second intercostal space.

**Figure 4:**
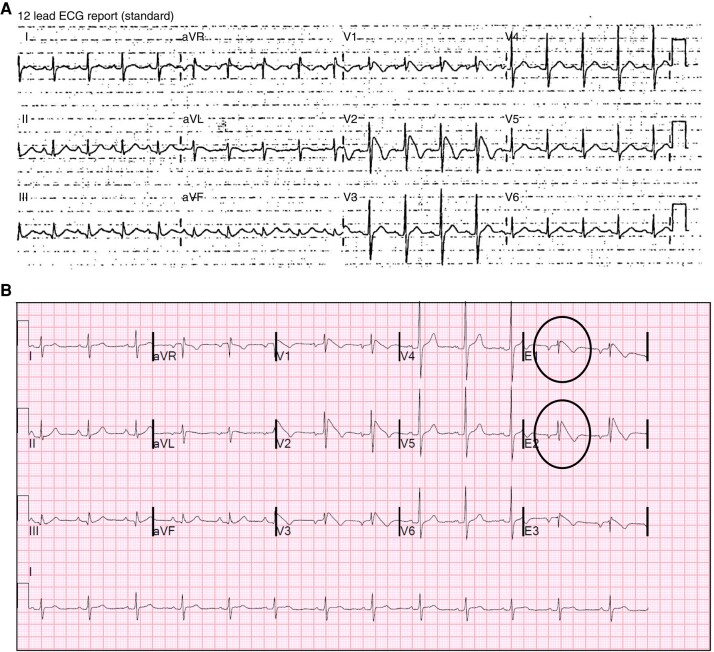
A 55-year-old Asian male with fever-induced type 1 Brugada electrocardiogram (ECG) pattern in leads V1 and V2 recorded in the fourth intercostal space (*A*), which is also present on a follow-up 12-lead high precordial ECG (*B*); in the latter leads, V3 represents lead V1 recorded in the third intercostal space, E1 is V2 recorded in the third intercostal space, E2 is V1 recorded in the second intercostal space, and E3 is V2 recorded in the second intercostal space.

All 358 subjects had at least 2 tracings recorded, of which at least 1 was a resting 12-lead or HPL 12-lead ECG. In total, 1651 resting/HPL ECGs (median 4, range 1–14) and 621 HPL Holter recordings from 269 subjects (median 2, range 1–8) were available; among these, 535 ECGs and 20 HPL Holter recordings were not available for direct review but adequately described in the medical notes. Median time between resting ECG recordings was 413 days [IQR 228], 355 [IQR 294] in Group 1 and 424 [IQR 204] in Group 2 (*P* = NS). Median time between HPL Holter recordings was 623 days [IQR 430], 546 [IQR 389] in Group 1 and 637 [IQR 436] in Group 2 (*P* = NS). Over a median follow-up of 72 months (IQR 75), 42/77 (55%) subjects in Group 1 showed a spT1 in at least 1 repeat resting HPL ECG or HPL Holter recording; of these, 5 had a fever-induced T1 at presentation. No subjects in this group showed a persistent spT1 in all the available tracings. In Group 2, 36/281 subjects (13%) had a newly detected spT1 (1.9 per 100 person-year); this was evident on a follow-up resting HPL ECG in 10/281 cases (3.5%), during the active or recovery phase of an exercise tolerance test in 3/281 cases (1%), and only on HPL Holter recordings in 23/281 subjects (8%); in this latter group, the spT1 was detected on the first HPL recording performed as part of the initial clinical evaluation within 30 days of the first consultation in 10 subjects (43%) and during a follow-up recording in 13 cases. None of the subjects declared use of drugs known to potentially unmask the spT1 pattern or high temperature at the time of the recording. The median time at new spT1 detection was 12 months [IQR 41], with 25/36 (70%) cases being detected within 1 month from the first evaluation at our centre. *Figures 2–4* show examples of spT1 occurring at follow-up.

Those patient from Group 2 with a newly detected spT1 pattern were predominantly males, older at first presentation, and less likely to have a familial history of sudden cardiac death, whereas there were no differences in ethnicity, proband status, genetic background, and previous AEs when compared to patient from Group 2 without a spT1 pattern (*Table 2*). There were no electrocardiographic predictors able to identify subjects from Group 2 who showed a new spT1 pattern during follow-up (*Table 3*). The clinical features of patients showing a spT1 at presentation or at HPL monitoring are detailed in *Table 4*.

**Table 2:** Summary of demographic and clinical characteristics of BrS patients with a newly identified spontaneous BrS type 1 vs. those with a consistently concealed BrS type 1 pattern

	G2 SpT1 at follow-up = 36	G2 no spT1 = 245	*P* value/odds ratio
Male gender (%)	25 (71)	102 (41)	***P* = 0.002/OR 3.18 (CI 1.15–6.7)**
Age at presentation	48 [IQR 16]	43 [IQR 26]	**0.030**
White ethnicity (%)	26/32	190/225 (84)	NS
Proband status (%)	27/35 (77)	137/245 (56)	NS
SCN5A +	8/28 (29)	24/119 (20)	NS
aCA/documented pVT	2/36 (6)	14/245 (6)	NS
Family history of SD	9/36 (25)	144/240 (60)	***P* = 0.001/OR 0.27 (CI 0.13–0.61)**

Values are expressed as median with IQR or absolute number with percentage. Bold values are only for significant *P* values.

aCA, aborted cardiac arrest; pVT, polymorphic ventricular tachycardia; SD, sudden death.

**Table 3:** Baseline ECG characteristics in BrS patients with a newly identified spontaneous type 1 pattern vs. those with a consistently concealed BrS type 1 pattern

	G2 spT1 at follow-up = 36	G2 no spT1 = 245	*P* value
QRS duration	102 [18]	100 [18]	NS
RR interval	861 [233]	845 [194]	NS
PR interval	170 [45]	166 [34]	NS
QTc	418 [33]	424 [28]	NS
First-degree AV block	6 (16%)	37 (15%)	NS
QRS > 110** **ms (%)	11 (31)	68 (28)	NS
S-wave in lead I > 40** **ms and/or >100 µV (%)	23/36 (64)	119/241 (49)	0.06
fQRS (%)	0	12 (5)	NS
ERP (%)	6/34 (18)	36/226 (16)	NS

Values are expressed as median with IQR or absolute number with percentage.

Bold values are only for significant *P* values.

ERP, early repolarisation pattern; fQRS, fragmented QRS.

**Table 4:** Summary of demographic and clinical characteristics of BrS patients with a spontaneous type 1 pattern on presenting ECG vs. a newly identified spT1 on HPL Holter

	G1 spT1 at presentation ECG = 77	G2 SpT1 at HPL Holter = 23	*P* value
Male gender (%)	57 (74)	15 (65)	NS
Age at presentation	45 [17]	53 [20]	**0.04**
Proband status (%)	72 (94)	(65)	**< 0.001**
SCN5A +	18/55 (33)	7/19 (37%)	NS
Prior symptoms			
** **aCA/pVT	0^a^	0	NS
** **Syncope/pre-syncope	32 (42)	3 (13)	0.01
** **Palpitations	15 (19)	5 (21)	NS
Family history of SD	15/74 (20)	6/22 (27)	NS

Values are expressed as median with IQR or absolute number with percentage.

Bold values are only for significant *P* values.

^a^Two subjects had ventricular arrhythmias recorded.

aCA, aborted cardiac arrest; HPL, high precordial lead; pVT, polymorphic ventricular tachycardia; SD, sudden death.

**Figure 5:**
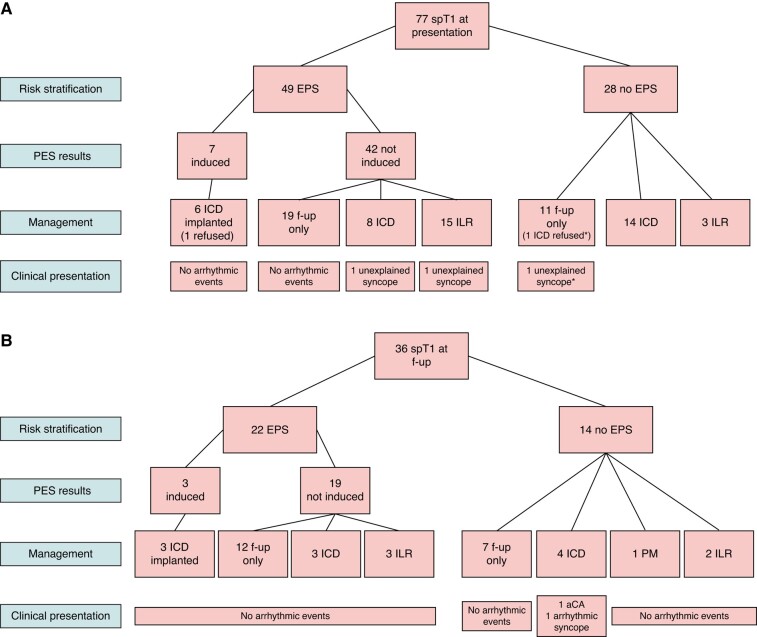
Symptoms at presentation and therapeutic strategy in subjects showing spontaneous type 1 (spT1) Brugada pattern, both at presentation (Panel A) and during follow-up (Panel B). EPS, electrophysiological study; ICD, implantable cardioverter defibrilator; ILR, implantable loop recorder.

Among the 102 ECGs with a spT1 that were available for review, we observed the spT1 in the standard fourth ICS only in 22 (22%), in the HPL only in 35 (34%), and in both configurations in 45 (44%). Among the 47 HPL Holter recordings with a spT1 for which the full disclosure recording was available for review, only in 1 (2%) the spT1 was confined to the standard fourth ICS, whereas in the remaining 98%, it was evident across the third and second ICS as well. From the HPL recordings available for full review, the median time in which a subject showed a spT1 was 326 min (IQR 1280) in Group 1 (data on 30 recordings) vs. 59 min (IQR 455) in Group 2 (data on 17 recordings), *P* = 0.004. A spT1 was observed in atypical leads in 10/102 (10%) 12-lead ECGs directly available for review (8 in aVR, 2 in aVL). Of the 77 subjects with spT1 at presentation in G1, 49 underwent programmed electrical stimulation, which induced ventricular fibrillation (VF) in 7. Of the 36 subjects in G2 who showed a spT1 during follow-up, 21 underwent programmed electrical stimulation, which induced VF in 3. *Figure 5* details their clinical presentation and management.

### Follow-up and outcomes

**Figure 6:**
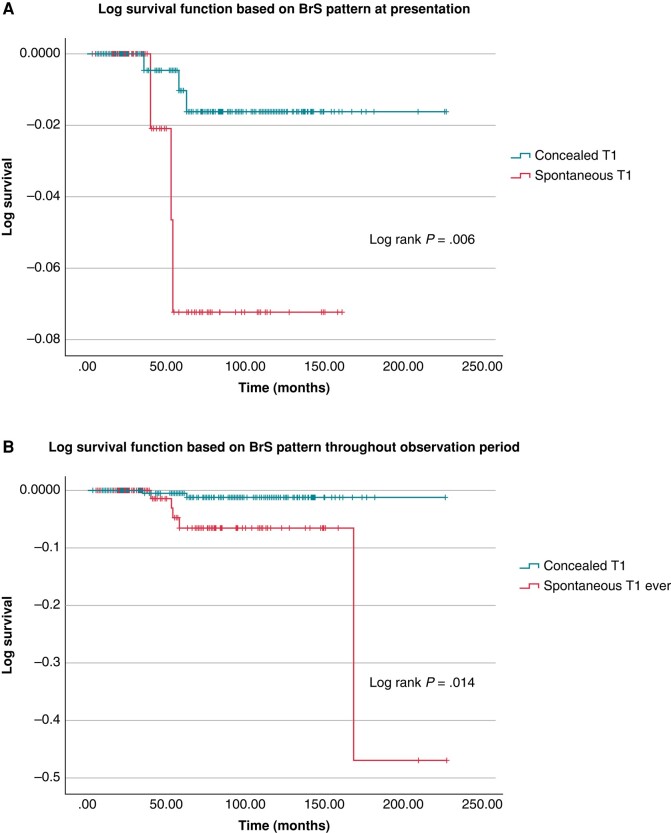
Kaplan–Meyer survival estimate based on spontaneous type 1 Brugada pattern presence throughout the follow-up period (*A*) or present at initial presentation (*B*). BrS, Brugada syndrome.

At follow-up, 12 patients experienced AEs (Graphical Abstract). To evaluate the association between a pre-existing or newly diagnosed spT1 and AE, we considered only subjects without previous aCA/documented VT and not while on prophylactic antiarrhythmic therapy with quinidine. Among these 342 subjects, over a median follow-up of 73 months, 4 subjects died for non-arrhythmic causes, 3 subjects experienced SCD, 3 others had appropriate ICD shock on VT/VF, and 1 had syncope with documented ventricular standstill recorded by an implantable loop recorder (this subject has been previously described^2^) and underwent ICD implantation. Of these 7 subjects with cardiac dysrhythmias, 5 had a spT1 pattern documented, 4 on the presenting ECG and 1 on the HPL Holter. The remaining 2 subjects had diT1 BrS pattern only; 1 is a white male referred at the age of 38 for syncopal episodes and family history of BrS in his father. He had negative PES and underwent ILR implantation, which showed episodes of ventricular standstill associated with syncope, for which he received an ICD. The other subject, white male, experienced SCD in his sleep at the age of 38; he was referred due to family history of BrS and juvenile SCD in his brother. His HPL Holter ECG showed a borderline, non-diagnostic spT1. The crude annual event rate was 1.15% in subjects with a spT1 at presentation, 0.52% in those with spT1 at follow-up, and 0.05% in those never showing a spT1; however, the difference between the two rates in the first two groups, considering the total person-years of follow-up (exact Poisson method), was not statistically different (*P* = 0.5). The total follow-up duration was not statistically different between the two groups. Survival analysis using the Kaplan–Meyer method showed that a spT1, occurring both at presentation and during lifetime, was associated with worse outcome (*Figure* *6A* and *B*). Univariate models showed that a spT1-BrS pattern was consistently associated with increased risk of events [spT1 at presentation: HR 6.3, 95% confidence interval (CI) 1.4–28, *P* = 0.016; spT1 at follow-up: HR 3.1, 95% CI 1.3–7.2, *P* = 0.008]. Multivariate analysis did not identify variables significantly associated with events.

## Discussion

This is the first study consecutively assessing the yield of repeat standard/HPL 12-lead ECGs and HPL Holter recordings in a large cohort of BrS patients over a long follow-up (median 6 years). Previous studies using resting 12-lead ECGs have highlighted that only a minority of BrS patients show a persistent spT1 (2–5%),^8^ and a substantial proportion (up to one-fifth) of those with a concealed T1 at presentation develop a spT1 pattern during follow-up.^8,9^ However, these studies investigated small cohorts and for a shorter period of time. Similarly, previous limited experience with HPL Holter recordings suggested that a new, transient spT1 can be identified in up to 30% of subjects initially labelled as ‘concealed’.^10,11^ One of the main findings of our study is that the combined use of resting HPL 12-lead ECGs and HPL Holter recordings can increase the detection of a transient spT1 pattern in up to 13% of subjects with a concealed T1 pattern at presentation over a long-term follow-up. The use of HPL Holter recordings increased the yield by 8% (23/281 subjects with diT1 were diagnosed based on the HPL Holter tracings), and, in almost half of the subjects, this occurred as part of the first clinical evaluation, within 30 days since being referred to our clinic. These results suggest that ambulatory ECG monitoring exploring the high right precordial leads should be considered a fundamental component of the initial evaluation of subjects with a suspicion of BrS, as it may indicate higher risk and avoid unnecessary SCB provocation tests, in light of recent concerns on the sensitivity of SCB provocation tests and the rate of false positive results.^14^ Unfortunately, 12-lead HPL Holter recordings are not available in many hospitals; therefore, patients with suspected or confirmed BrS pattern should be referred to specialized tertiary centres that are more likely to have such investigation tools. Where these are not available, repeated standard and HPL 12-lead ECGs, preferably recorded at rest and during effort^15^ at different times of day, remain the only option to investigate the presence and the burden of spT1 pattern. Another interesting finding from this study is that 5/12 (41%) of subjects with fever-induced T1 at presentation showed a dynamic spT1 during follow-up, in the absence of a reported increase in body temperature. It is recognized that fever may trigger or exacerbate arrhythmic manifestations of BrS, although a previous study has shown a 2% prevalence of T1 pattern in subjects with fever attending an emergency department who did not experience significant AE over a follow-up of 30 months.^16^ Whether the presence of a spT1 pattern in the absence of high temperature confers a higher risk in this sub-population could not be ascertained in this study due to the small sample size. Finally, we observed that patients with a spT1 pattern on initial assessment tended to be older, although this difference was not statistically significant. Whether this represents a specific evolution of the dynamicity of the spT1 pattern with age would require further research.

### Predictors of spontaneous type 1 pattern at follow-up

We tested several clinical and ECG parameters to evaluate their association with the development of a spT1 at follow-up; however, aside from male sex, no clear relationship was found (*Table 2*). This may reflect a more important role of transient, modulating factors such as sympatho-vagal balance and hormone levels, compared to more ‘fixed’ electrophysiological substrate characteristics; however, this hypothesis needs to be tested specifically.

### Clinical implications

Risk stratification in BrS is still a hot topic of debate. Several ECG traits have been associated with AEs, and intuitively, depolarization and/or repolarization abnormalities may underlie a more diseased substrate, which in turn would be more likely predisposing to arrhythmias^7^; however, the majority of these ECG markers failed to reliably predict the risk when applied to external populations, although more convincing results seem to arise when they are combined together.^7,17^ One of the most reliable markers of risk is the presence of a spT1 pattern detected at presentation, which consistently demonstrated to confer an increased risk of AEs during follow-up in the largest case series and metanalyses^6,18,19^; in fact, a spT1 at presentation is associated with an estimated three-fold increase in the annual event rate on average, compared to subjects with concealed or diT1 pattern at presentation (0.88% vs. 0.29% in asymptomatic subjects; 4.08% vs. 1.34% in those with previous syncope/aCA).^14^ We focussed our survival analysis on subjects who were asymptomatic at diagnosis (*Graphical Abstract*), as this is the group in whom risk stratification is more challenging; asymptomatic subjects with diT1 who never show a spT1 at follow-up carry a very low yearly risk of events (0.05%), as supported by other registry data.^12^ Conversely, as expected, the presence of a spT1 at presentation was associated with increased risk (HR 6.3); in addition, our data show for the first time that a spT1 detected during follow-up may increase the risk of AEs, although the number of subjects and events is low in this cohort (HR 3.1). Therefore, these results require validation in a larger cohort of subjects with spT1 during follow-up. Together, our findings provide important insights into the dynamicity and significance of a spT1. This clearly advocates for continuous reassessment of subjects with concealed T1 pattern at presentation, including the use of HPL Holter recordings. In fact, these may increase the identification of a suspicious or diagnostic BrS pattern and, together with new promising artificial intelligence (AI) ECG machine learning models,^20^ can reduce the need of pharmacological testing to confirm the diagnosis. In addition, HPL Holter monitoring has the potential to better define the individual risk, for example in subjects with unexplained or clearly arrhythmic syncope.^21^ Unfortunately, recent data suggest that considerable heterogeneity still exist among European countries with regard to the availability of specialized inherited cardiac condition clinics that can deliver this approach to the management of patients with BrS.^22^ There is no definitive data yet on the risk estimation associated with individual spT1 burden. Assuming the spT1 (at any time) is necessary from a pathophysiological point of view for the development of VF, based on current assumptions, at least one-third of our concealed diT1 cohort should have shown a spT1 at follow-up.^23^ This was, however, lower in our study (13%) and may reflect a higher proportion of lower risk cases: non-probands referred due to a family history of SCD. It is also possible that different genetic substrates (single-gene *SCN5A* variants vs. polygenic contribution) influence the ECG phenotype variability in BrS; whether this could also have an influence in arrhythmogenesis and potential for tailored therapeutic strategies needs to be elucidated in future research.^24^ Multivariate analysis using clinical and demographic parameters failed to find significant associations with events due to the low event rate.

## Conclusions

A spT1 Brugada pattern is a recognized marker of risk of life-threatening arrhythmias. However, ECG-based risk stratification in BrS remains challenging and complicated by the fluctuations of the ECG. This study confirms the importance of repeat ECG evaluation and the significant added value of HPL 12-lead 24-h ambulatory monitoring in identifying subjects with a transient spT1 pattern. This has implications for estimating risk in this population.

### Study limitations

Due to the retrospective, non-controlled nature of this study, the follow-up appointment intervals were not homogeneous in the study cohort; hence, some patients may have had a substantial higher number of resting and ambulatory ECGs recorded; HPL Holter recordings were not available for all subjects; exercise tolerance test results were not available for all subjects and not performed exploring the HPLs. These factors may have influenced the real prevalence of a dynamic spT1 in the study. The risk of AEs in subjects with spT1 at follow-up requires validation in a larger cohort of subjects with spT1 during follow-up. However, the results from this real-life, population-based cohort of BrS patients offer important insights on the actual prevalence of spT1 and its prognostic implications over a long-term follow-up.

## Data Availability

The data underlying this article cannot be shared publicly due to participant privacy. Some data can be shared upon reasonable request to the corresponding author.
